# Control of fault plane geometry on the formation of a normal fault-related anticline: an experimental approach

**DOI:** 10.1038/s41598-017-00249-x

**Published:** 2017-03-13

**Authors:** Wei Long, Zhongquan Li, Ying Li, Junliang Chen, Hongkui Li, Shuangshuang Wan

**Affiliations:** 1 State Key Laboratory of Oil and Gas Reservoir Geology and Exploitation, Chengdu University of Technology, 1 Dongsanlu, Chengdu, 610059 China; 2 Key Laboratory of Tectonic Controls on Mineralization and Hydrocarbon Accumulation, Ministry of Land and Resources, Chengdu University of Technology, 1 Dongsanlu, Chengdu, 610059 China; 3 School of Geosciences, King’s Colleges, University of Aberdeen, AB24 3UE Aberdeen, UK; 4 Exploration and Development Research Institute of Daqing Oilfield Company Ltd., Daqing, 163712 China

## Abstract

In one of the largest oil-gas fields in Daqing, China, the anticlines are important structures that hold natural gas. The origin of the symmetric anticlines, which have bends on both the limbs, remains under debate. This is especially true in the case of the anticline in Xujiaweizi (XJWZ), which has recently been the focus of gas exploration. A compressive force introduced by a ramp/flat fault was suggested as its origin of formation; however, this is inconsistent with the reconstruction of the regional stress fields, which show an extensive environment. An alternative explanation suggests a normal fault-related fold under extensive stress. However, this mechanism has difficulty explaining the very localized, rather than wide-spread, development of the anticline along the proposed controlling normal fault. The well-developed bends on both limbs of the anticline are also very different from the typical roll-over anticline. Here, we conduct an experimental study showing that the very localized development of the bent-on-both-limbs anticline is controlled by the geometry of the underlying fault-plane. A ramp/flat fault plane can introduce an anticline with bends on both limbs, while a smooth fault plane will develop a roll-over anticline with a bend on only one limb.

## Introduction

**Figure 1:**
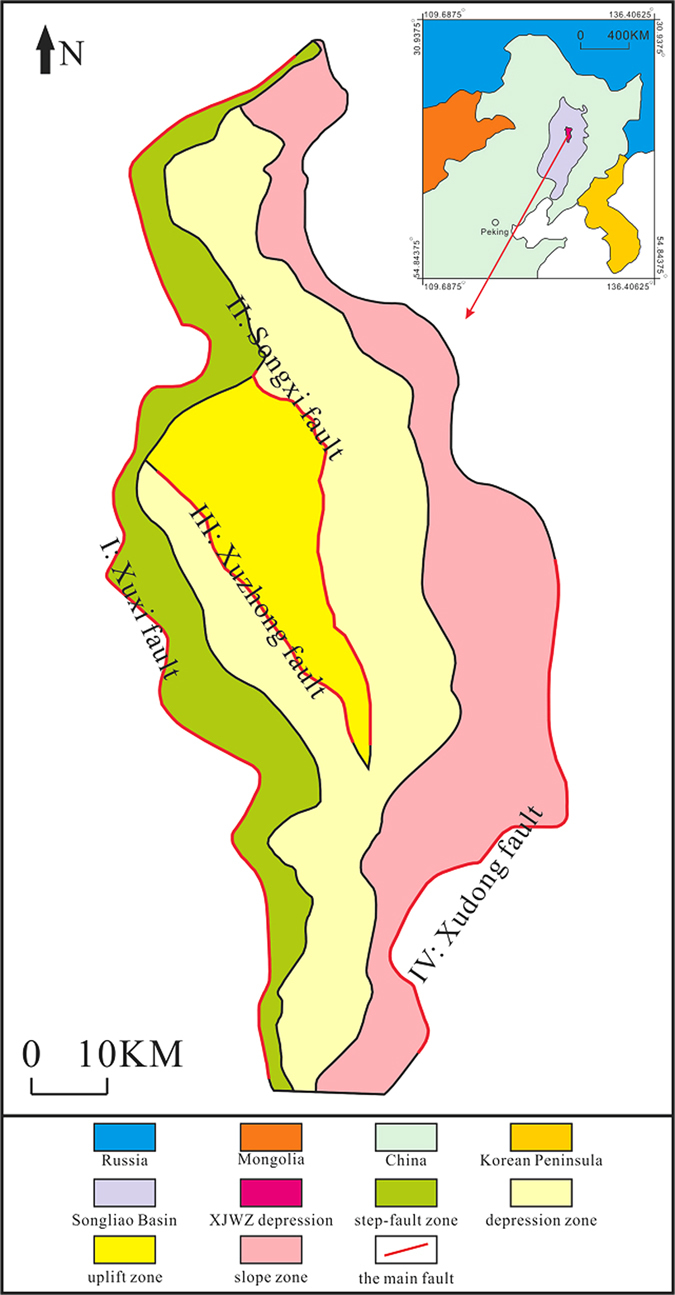
A tectonic map showing the major fault systems related to the XJWZ depression (adapted from Figs 2–14 presented by Ren^19^). The Xuxi fault, which is on the west boundary of the XJWZ depression, is responsible for the fault-related anticline. The data are from http://www.265.me/, and the figures were produced using the CorelDRAW software (Version X8) (http://www.corel.com/cn/). The inset shows the geographical location of XJWZ, which is adapted from Figure 2–3 presented by Zhang^1^.

**Figure 2:**
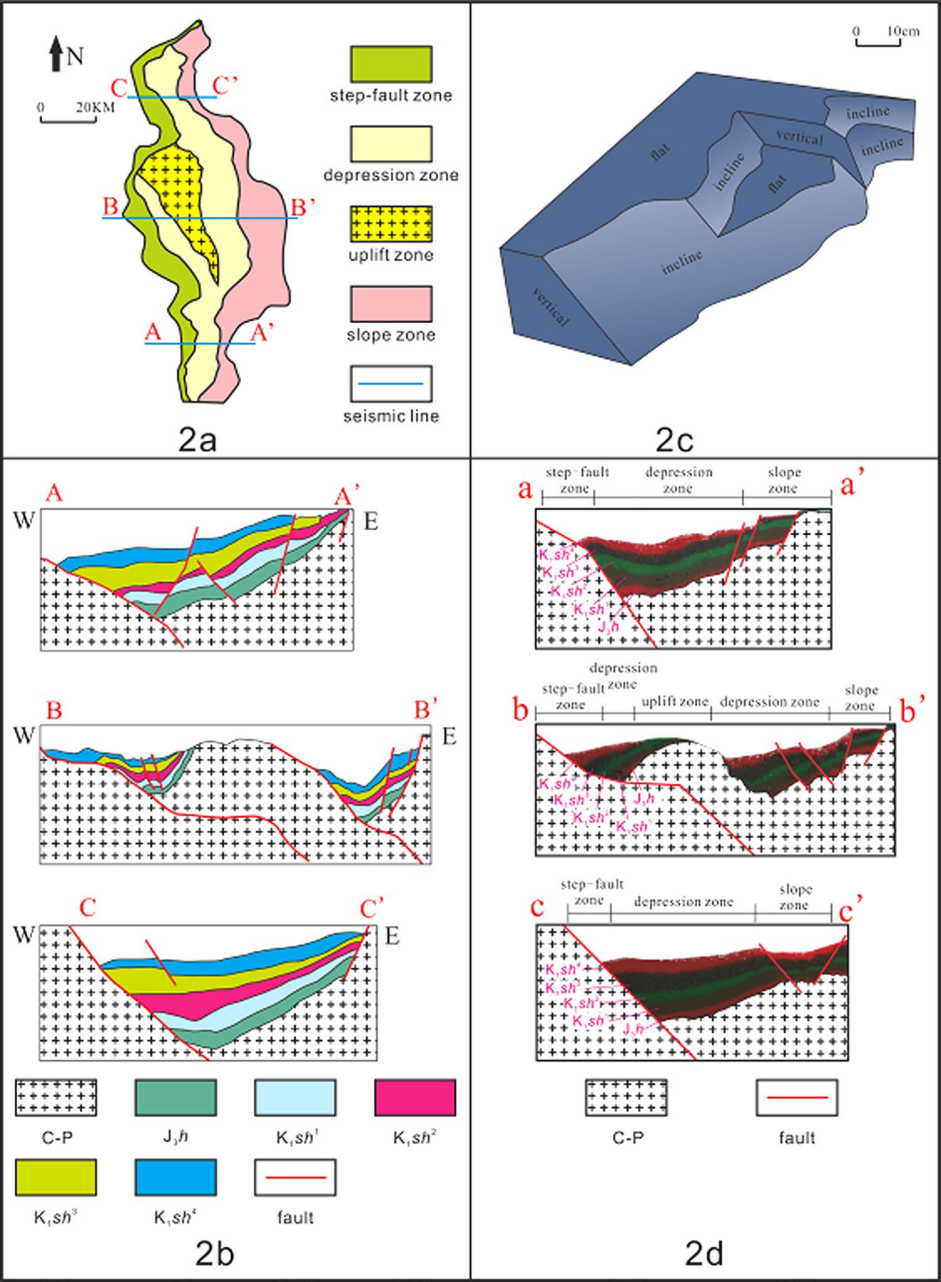
(**a**) The tectonic map of the XJWZ depression showing the controlling normal fault to the west (adapted from Figs 2–14 presented by Ren^19^). (**b**) The seismic observation showing the development of a symmetric anticline in the middle of the XJWZ depression. (**c**) The shape of basement used in the 3-D simulation box. (**d**) The result of the 3-D simulation. The figures were produced using the CorelDRAW software (Version X8) (http://www.corel.com/cn/).

The anticline in Xujiaweizi (XJWZ) is the focus of recent natural gas exploration in Daqing (Fig. 1)^1^, one of the largest oil-gas fields in China^2^. Deciphering the mechanism of the formation of the XJWZ anticline is of great importance for the understanding of the production, transportation, and preservation of the natural gas that it holds^3, 4^. It has been suggested that the XJWZ anticline was produced under compressive stress^5, 6^. However, at the beginning of the Late Triassic period, spreading occurred in XJWZ^3^ (Table 1). The reconstruction of the regional stress background at the time of the anticline’s formation indicates extensive environments^7^. In contrast, the XJWZ anticline may be related to the normal fault to its west (Fig. 2a). However, the anticline only formed in the middle of the XJWZ depression, and no anticline with a well-developed geometry can be observed in the adjacent region along the proposed controlling fault (Fig. 2b). More importantly, the bends on both limbs of the XJWZ anticline are well developed (Fig. 2b), which differs from the typical roll-over anticline in which the bends are developed on the limb that is associated with the controlling fault.

**Table 1:** Stratigraphic chart in XJWZ depression.

System	Series	Group	Symbol	Tectonic stage
Cretaceous	Lower	Quantou	K_1_ *q*	Depression stage
		Denglouku	K_1_ *d*	
		Yingcheng	K_1_ *y*	Fault-depression inversion stage
		Shahezi	K_1_ *sh*	Strong rifting stage
Jurassic	Upper	Huoshiling	J_3_ *h*	Initial rifting stage
Basement	C-P

It has been noted that the geometry of the normal fault-related folds is associated with the shape of the underlying fault plane^8, 9^. The fault plane under the XJWZ anticline is characterized by a ramp/flat shape^3^ (Fig. 2b), while the fault planes to the north and south are much smoother (Fig. 2b). Thus, the geometry of the underlying fault plane may have controlled the formation of the XJWZ anticline.

This work applies experimental simulations to decipher the role of the underlying fault shape in the geometry of the overriding folds. Physical simulations of the geological structure have been widely used to study geological evolution^10–13^. A 3-D simulation with a boundary condition that is similar to the XJWZ depression was conducted to examine the response of the fold geometry to the underlying plane shape. A 2-D simulation was also conducted to reveal the dynamic processes associated with the formation of the anticline on a ramp/flat fault plane. The simulated results show consistency with the observed structure in the XJWZ depression, which provides rather robust evidence for the control of the underlying fault shape on the formation of the XJWZ anticline.

## Method

The physical experiments were conducted in boxes based on the principle of similarity^14–16^. In the experiments, a strata thickness of 100 m in the real world is equivalent to a 1 cm layer of sand. Consequently, the sizes of the boxes were determined to be 60 cm × 30 cm × 16 cm and 50 cm × 60 cm × 16 cm for the 2-D and 3-D simulations, respectively.

**Figure 3:**
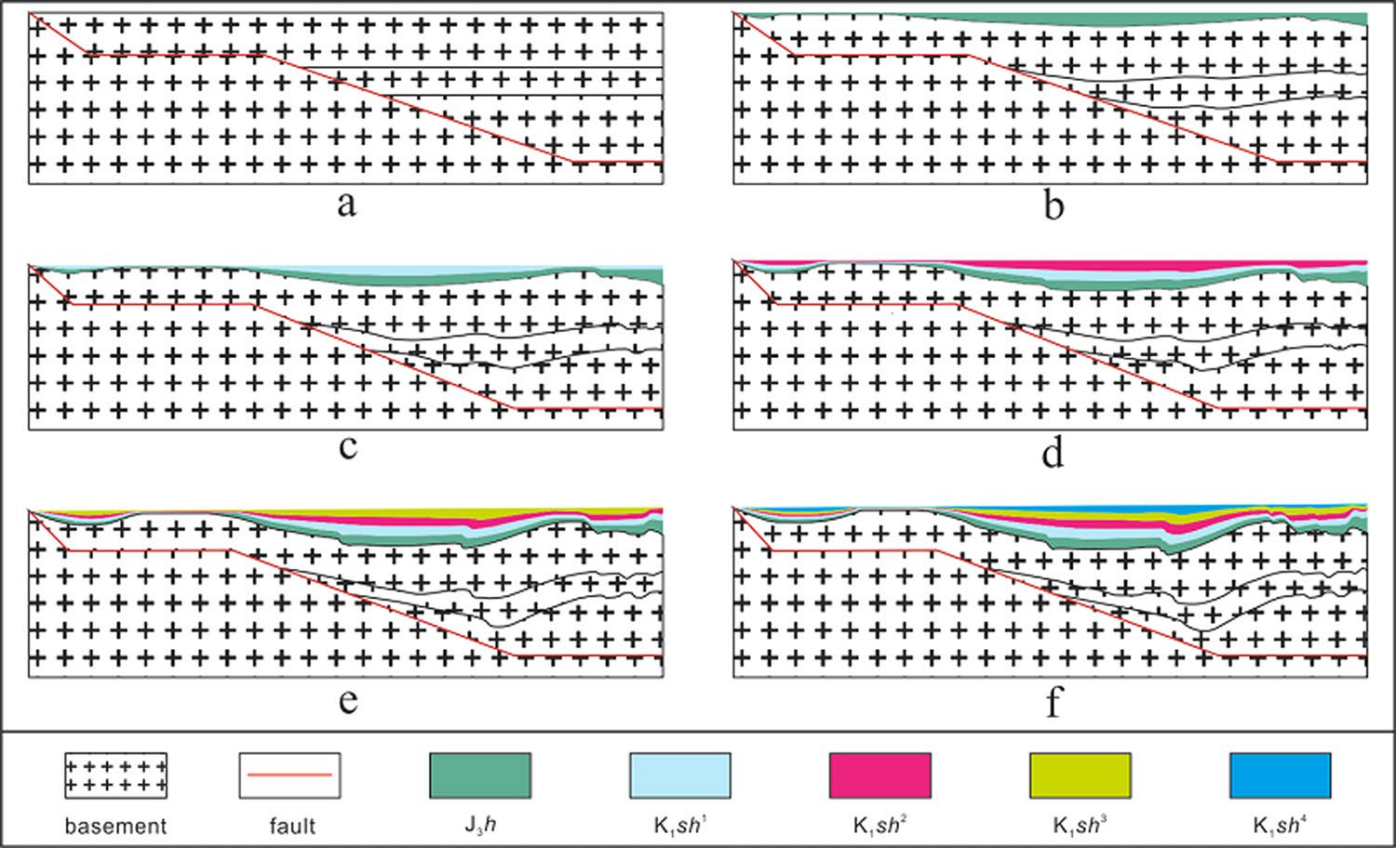
The result of the 2-D simulation showing the dynamic processes corresponding to the formation of the bends on both limbs of the anticline with a ramp/flat basement shape.

Three layers were included in the simulation box. The bottom layer is a fixed basement, and the shape of its upper boundary mimics the fault plane (Fig. 3). The 2-D model adopts a ramp/flat shape that mimics the fault plane under the XJWZ anticline (Fig. 3). The 3-D model uses a real shape revealed by the seismic imaging, which has a ramp/flat shape in the centre and a smooth slope on both sides of the box (Fig. 2c).

The upper layer is composed of dyed dry quartz sand with a particle size of 0.3–0.45 mm. The deformation of the dry quartz sand, which has an internal friction angle of approximately 31° and an internal friction coefficient of approximately 0.55, meets the Mohr-Coulomb fracture criterion and is very similar to the brittle deformation behaviour of the sedimentary rocks in the shallow crust (<15 km)^17, 18^. Thus, the dry quartz sand can be used to simulate the deformation in the XJWZ depression. The quartz sand is dyed with different colours for different layers to allow the deformation to be visually observed. The layers of quartz sand were continuously filled into the space created by the extension during the simulation (Fig. 3).

Between the fixed basement and the quartz sand layers is a rubber belt that can be extended uniformly in the 2-D simulation and a canvas sheet in the 3-D simulation. In the 2-D simulation, the rubber belt was fixed on the walls that align with both sides of the boxes (Fig. 3). The wall on the side of the basement is fixed, while the wall on the other side moves outward with a speed of 0.025 mm/s. The overall distance of the movement is 10 cm. In the 3-D simulation, the canvas sheet is only fixed to the outward moving wall, which shifts at a speed of 0.025 mm/s and has a moving distance of 6 cm.

A glassy side wall was added to the 2-D simulation to record the dynamic deformation history of the dyed quartz sand under the extensive force by a digital camera. The final result of the 3-D simulation is revealed by slicing the box into sections.

The similarity of the key factors between the simulation and real conditions can be found in Table 2. The main difference is caused by the thickness of strata (0.16 m vs. 1600 m) and the velocity of the lateral extension (2.5 × 10^−5^ m/s vs. 2.46 × 10^−11^ m/s). Thus, the calculated vertical stress and the vertical strain rate differ greatly (Table 2).

**Table 2:** Calculation of experimental similarity.

Parameter	Symbol	Unit	Experimental value	Natural value	Similarity^*^
Thickness	h	m	0.16	1600	0.0001
Density	ρ	kg·m^−3^	1297	2800	0.46
Gravity acceleration	g	m·s^−2^	9.81	9.81	1
Speed	v	m·s^−1^	2.5E−05	2.46E−11	1.02E + 6
Vertical stress	σ = ρgh	Pa	2035.77	4.39E + 7	4.64E−5
Vertical strain rate	ε = v/h	s^−1^	1.56E + 4	1.54E−14	1.02E10

^*^Calculated from the ratio between experimental value and natural value.

## Results and Discussion

The simulation shows the development of a symmetric anticline with bends on both its limbs in the middle of the experimental box, where the basement has a ramp/flat shape (Fig. 2d). In contrast, typical roll-over anticlines, with a bend on one limb, were observed where the basement has a smooth slope (Fig. 2d). The similarity between the simulated and the observed structures of the anticlines in the XJWZ depression collectively suggests control by the fault plane shape of the geometry of the overriding anticline.

The dynamic processes that correspond to the formation of the symmetric anticline on the ramp/flat fault plane have been observed in the 2-D simulation (Fig. 3). The extension produced space on the plane slopes on both sides of the plane platform. Thus, the strata tend to bend downward due to the gravitational force associated with the space created by the extension. In contrast, the strata on the platform remain stable. Thus, downward bends develop on the slopes of both sides of the plane platform, which creates an anticline with bends on both of its limbs.

## Conclusion

The combination of the experimental simulation and the seismic observation suggests that the formation of the symmetric anticline with bends on both its limbs may be related to the ramp/flat shape of the underlying fault plane. This very unusual control by the plane shape of the geometry of the overriding anticline may explain the very localized development of the symmetric anticline in the XJWZ depression that acts as an important gas holding reservoir. Future explorations of the natural gas reservoir in this region may benefit from identifying a fault plane with a ramp/flat shape.
